# Critical Limitations in Comparing ChatGPT and DeepSeek for Orthopedic Assessment

**DOI:** 10.2196/90242

**Published:** 2026-03-17

**Authors:** Orhan Ayas, Alaeddin Acar

**Affiliations:** 1 Department of Orthopedics and Traumatology, Fethi Sekin City Hospital, Elazığ, Turkey; 2 Department of Neurosurgery, Kulu State Hospital, No 4, 139518 Street, Dinek, Kulu, Konya, 42770, Turkey, 90 542 472 37 23

**Keywords:** ChatGPT, large language model, LLM, orthopedic, multiple-choice question, MCQ

We read with great interest the study by Anusitviwat et al [1], which compared the performance of ChatGPT and DeepSeek in orthopedic examinations. While the study provides timely insights into the utility of large language models (LLMs) in medical education, we identified specific methodological and terminological limitations that warrant clarification to ensure the validity and reproducibility of the findings.

## Misinterpretation of Reliability Statistics

The authors state that the “interrater reliability between the two LLMs” was evaluated using the Cohen κ coefficient [1]. Mathematically, measuring the agreement between two independent raters (interrater) yields a single coefficient. However, the results report two separate values: κ of 0.81 for ChatGPT and κ of 0.78 for DeepSeek [1]. This finding, combined with the methodology stating that questions were input on “separate days” [1], indicates that the study actually measured intramodel consistency (test-retest reliability) rather than the agreement between the models. Labeling internal consistency as “interrater reliability” is terminologically inaccurate and misrepresents the statistical relationship between the two models.

## Linguistic Ambiguity and Generalizability

The manuscript does not specify the language of the input multiple-choice questions (Thai or English) used in the assessments. This omission is critical, as the impact of input language on LLM performance is well-documented. For instance, Noda et al [2] demonstrated that GPT-4V’s accuracy on the Japanese Otolaryngology Board Examination significantly improved from 24.7% (Japanese input) to 47.3% when translated into English. This finding underscores that models optimized for English exhibit distinct performance disparities in non-English languages. Without clarifying whether the assessments were administered in the local language or English, it is impossible to determine if the reported accuracy gap between ChatGPT (80.4%) and DeepSeek (74.2%) stems from medical reasoning capabilities or linguistic processing proficiency.

## Reproducibility and Interface Transparency

The methodology reports the use of “Reason” and “DeepThink” functions but does not explicitly state whether the models were accessed via web-based user interfaces or application programming interfaces [1]. This distinction is vital for reproducibility. Web-based user interfaces are subject to opaque updates and lack the stability of controlled application programming interface environments. Without defining the access method and the specific prompt structures used, the experimental conditions cannot be replicated.

## Risk of Data Contamination

The authors note that the multiple-choice questions “have been used in orthopedic examinations for more than 5 years.” This longevity significantly increases the risk of data contamination, as older items likely exist in public repositories within LLM training corpora, potentially conflating memorization with reasoning. To ensure validity, recent benchmarks use private datasets (Busch et al [3]) or questions postdating the model’s training cutoff (Noda et al [2]). The absence of such controls in this study undermines the internal validity of the comparison.

## Data Reporting Discrepancy

Finally, we noted a minor discrepancy in Table 2. In the “Pelvic and spine injury” category (n=19), the accuracy for the Reason function is listed as 16 (68.8%) [1]. Mathematically, 16 of 19 corresponds to approximately 84.2%, not 68.8%. We respectfully invite the authors to clarify this value to ensure the precision of the tabulated data.

## Abbreviations

LLM: large language model
